# Editorial: Amino Acid Transport and Metabolism During Homeostasis and Inflammation

**DOI:** 10.3389/fimmu.2021.833258

**Published:** 2022-01-17

**Authors:** Danay Cibrian, Francesc Baixauli, Manuel Palacin

**Affiliations:** ^1^ Vascular Pathophysiology Area, Centro Nacional de Investigaciones Cardiovasculares (CNIC), Madrid, Spain; ^2^ Department of Immunometabolism, Max Planck Institute for Immunobiology and Epigenetics, Freiburg, Germany; ^3^ Institute for Research in Biomedicine (IRB-Barcelona) within the Barcelona Institute of Science and Technology (BIST), U731 CIBERER and Department of Biochemistry and Molecular Biomedicine, University of Barcelona, Barcelona, Spain

**Keywords:** amino acid transporter, SLC7A5, SLC3A2, SLC6A14 (ATB^0+^), L-trptophan, CAT1 cationic amino acid transporter

## Introduction

The solute carrier (SLC) protein families mediating amino acid uptake in human cells are SLC1, SLC3, SLC6, SLC7, SLC16, SLC17, SLC25, SLC32, SLC36, SLC38, and SLC43 (1). While SLC25 is a family of mitochondrial transporters and SLC17 and SLC32 mediate vesicular amino acid transport, the transporters of the other families are expressed in the plasma membrane (1). Besides their essential role as building blocks during protein synthesis, amino acids are an important energy source; they act as precursors for a variety of metabolites, and regulate important signaling pathways.

Elevated amino acid uptake is a common feature of activated immune cells (2–4), and cancer cells (5, 6), which sustain immune functions and malignancy, respectively. This Research Topic includes three literature review articles that focus on amino acid transporters in NK cells, myeloid cells and the gut epithelium. It also includes three original research articles describing the regulation of amino acid transporters during inflammation. Their major findings are highlighted below.

The SLC3A2 protein (CD98hc, 4F2hc) forms disulfide-bound heterodimers with members of the SLC7 family, including SLC7A5 (LAT1) (1, 7). The SLC7A5-SLC3A2 complex is responsible for the delivery of most of L-Leu to malignant as well as immune cells, thereby playing a major role in the regulation of the mTOR pathway (8). This complex works as an antiporter system mediating the cellular uptake not only of L-Leu but also of L-Phe, L-Trp, L-His, L-Met, and L-Tyr, while removing L-Gln from the cell. In addition, increased L-Gln transport by ASCT2 (SLC1A5) and SNAT1 (SLC38A1) is also a key feature of immune and malignant cells (9, 10). The expression of amino acid transporters, including SLC7A5-SLC3A2 and SLC1A5, is increased in several tumors, correlating with cancer progression (5). Cancer cells take up nutrients more efficiently than immune cells, thus generating a hostile, nutrient-depleted tumor microenvironment that suppresses the proper function of cytotoxic immune cells. This is a major hurdle to be overcome for the implementation of adoptive transfer of genetically engineered T cells or NK cells expressing chimeric antigen receptors (CARs) to treat cancer. The work of Nachef et al. suggests novel strategies to strengthen immunotherapy against cancer, focusing on positive and negative regulators of SLC7A5, SLC3A2 and SLC1A5 protein expression. Potential strategies to enhance the expression of the aforementioned SLC proteins in immune cells include overexpressing positive regulators, increasing mRNA stabilization, and promoting their surface localization or blocking their degradation (Nachef et al.).

Many cytokines upregulate the expression of amino acid transporters in activated immune cells. IL-18 upregulates the expression of the SLC7A5-SLC3A2 amino acid complex in NK cells, *in vitro* (11) (Khan et al.). However, the work of Khan et al. reveals that IL-18 signaling is dispensable for the upregulation of nutrient transporters during murine cytomegalovirus (MCMV) infection. This study demonstrates that the expression of the SLC7A5-SLC3A2 complex in NK cells is regulated by Myd88 signaling, and enhanced by IL-33 as well as IL-12 cytokines. The expression of this complex is higher in Ly49H+ NK subsets, suggesting that recognition of the MCMV protein m157 by the receptor Ly49H also enhances the expression of amino acid transporters *in vivo* (Khan et al.).

Functional polarization of myeloid cells is closely associated with the expression of enzymes metabolizing key amino acids, such as L-Arg. Halaby and McGaha described how amino acid availability and metabolism affect the function of two key populations of myeloid cells, macrophages and myeloid-derived suppressor cells. The work focuses on transport of several amino acids such as L-Arg, which enters macrophages through cationic amino acid transporters (CAT), L-Gln and L-Ser, which are transported by ASCT2 or SNAT1/2 carriers, and L-Trp and branched-chain amino acids, imported to the cells by the SLC7A5-SLC3A2 complex (Halaby and McGaha).

Amino acids are required for the activation of the mTOR signaling pathway (12). The work of Yang et al. showed that Taraxasterol (TAS) suppressed mTORC1 and mTORC2 activation induced by LPS in macrophages. The incubation of macrophages with TAS, as well as mTOR pathway inhibitors, effectively impaired LPS-induced NLRP3 inflammasome activation, highlighting its anti-inflammatory properties (Yang et al.). However, whether TAS controls amino acid uptake at any level remains unexplored.

ATB^0,+^ (SLC6A14) is an influx system for neutral and cationic amino acids, including L-Arg. This transporter is mainly expressed in the lung and the intestine (13–15). ATB^0,+^ activity in bronchial epithelial cells is modulated by inflammatory stimuli, such as LPS and TNFα, as well as by flagellin from *Pseudomonas aeruginosa* (FLA-PA) (16, 17). The study of Barilli et al. further explored the effect of FLA-PA on additional L-Arg transporters in human bronchial cells, including SLC7A1 (CAT1), SLC7A2 (CAT2A and CAT2B splice variants), SLC7A7 (y^+^LAT1) and SLC7A6 (y^+^LAT2). Their results suggest a clear specificity for ATB^0,+^ upregulation by FLA-PA, and reveal that induction of the SLC6A14 gene by flagellin directly depends on the activity of the TLR5/NF-κB signaling pathway (Barilli et al.).

L-Trp is an important source of metabolites that exert main functions in the gut, the skin, the nervous system, as well as the immune system (18). Host cells and microbiota metabolize dietary L-Trp into kynurenine (Kyn) and its derivatives 5-hydroxytryptophan (5-HT, serotonin) and tryptamine. Some Trp metabolites, both host- and microbe-derived, bind directly to the host transcription factor aryl hydrocarbon receptor (AHR), modulating its function (18). The work of Grifka-Walk et al. provides an overview of the amino acid carrier systems mediating uptake of dietary L-Trp in the intestine, as well as the function of its metabolites in the gut and beyond.

This Research Topic provides novel insights into the mechanisms regulating amino acid transporter expression and function during inflammation. The development of novel inhibitors for amino acid transporters represents a promising approach to fight chronic inflammation and cancer.

## Author Contributions

All authors listed have made a substantial, direct, and intellectual contribution to the work and approved it for publication.

## Funding

DC is supported by grant from Ayudas Fundación BBVA a Equipos de Investigación Científica (BIOMEDICINA-2018) and “La Caixa” Banking Foundation (HR17-00016). MP is supported by the Spanish Science, Innovation and University Ministry (RT2018—094211-B-100-FEDER), La Caixa Foundation (LCF/PR/HR20/52400017) and La Marató-TV3.

## Conflict of Interest

The authors declare that the research was conducted in the absence of any commercial or financial relationships that could be construed as a potential conflict of interest.

## Publisher’s Note

All claims expressed in this article are solely those of the authors and do not necessarily represent those of their affiliated organizations, or those of the publisher, the editors and the reviewers. Any product that may be evaluated in this article, or claim that may be made by its manufacturer, is not guaranteed or endorsed by the publisher.

## References

[1] Pizzagalli MD Bensimon A Superti-Furga G. A Guide to Plasma Membrane Solute Carrier Proteins. FEBS J (2021) 288(9):2784–835. doi: 10.1111/febs.15531 PMC8246967 32810346

[2] Sinclair LV Rolf J Emslie E Shi YB Taylor PM Cantrell DA. Control of Amino-Acid Transport by Antigen Receptors Coordinates the Metabolic Reprogramming Essential for T Cell Differentiation. Nat Immunol (2013) 14(5):500–8. doi: 10.1038/ni.2556 PMC3672957 23525088

[3] Loftus RM Assmann N Kedia-Mehta N O'Brien KL Garcia A Gillespie C. Amino Acid-Dependent Cmyc Expression Is Essential for NK Cell Metabolic and Functional Responses in Mice. Nat Commun (2018) 9(1):2341. doi: 10.1038/s41467-018-04719-2 29904050 PMC6002377

[4] Cibrian D Castillo-Gonzalez R Fernandez-Gallego N de la Fuente H Jorge I Saiz ML. Targeting L-Type Amino Acid Transporter 1 in Innate and Adaptive T Cells Efficiently Controls Skin Inflammation. J Allergy Clin Immunol (2020) 145(1):199–214.e11. doi: 10.1016/j.jaci.2019.09.025 31605740

[5] Broer S. Amino Acid Transporters as Targets for Cancer Therapy: Why, Where, When, and How. Int J Mol Sci (2020) 21(17):6156. doi: 10.3390/ijms21176156 PMC7503255 32859034

[6] Okano N Naruge D Kawai K Kobayashi T Nagashima F Endou H. First-In-Human Phase I Study of JPH203, an L-Type Amino Acid Transporter 1 Inhibitor, in Patients With Advanced Solid Tumors. Invest New Drugs (2020) 38(5):1495–506. doi: 10.1007/s10637-020-00924-3 32198649

[7] Palacin M Errasti-Murugarren E Rosell A. Heteromeric Amino Acid Transporters. In Search of the Molecular Bases of Transport Cycle Mechanisms. Biochem Soc Trans (2016) 44(3):745–52. doi: 10.1042/BST20150294 27284037

[8] Cormerais Y Giuliano S LeFloch R Front B Durivault J Tambutte E. Genetic Disruption of the Multifunctional CD98/LAT1 Complex Demonstrates the Key Role of Essential Amino Acid Transport in the Control of Mtorc1 and Tumor Growth. Cancer Res (2016) 76(15):4481–92. doi: 10.1158/0008-5472.CAN-15-3376 27302165

[9] Broer A Rahimi F Broer S. Deletion of Amino Acid Transporter ASCT2 (SLC1A5) Reveals an Essential Role for Transporters SNAT1 (SLC38A1) and SNAT2 (SLC38A2) to Sustain Glutaminolysis in Cancer Cells. J Biol Chem (2016) 291(25):13194–205. doi: 10.1074/jbc.M115.700534 PMC4933233 27129276

[10] Nakaya M Xiao Y Zhou X Chang JH Chang M Cheng X. Inflammatory T Cell Responses Rely on Amino Acid Transporter ASCT2 Facilitation of Glutamine Uptake and Mtorc1 Kinase Activation. Immunity (2014) 40(5):692–705. doi: 10.1016/j.immuni.2014.04.007 24792914 PMC4074507

[11] Almutairi SM Ali AK He W Yang DS Ghorbani P Wang L. Interleukin-18 Up-Regulates Amino Acid Transporters and Facilitates Amino Acid-Induced Mtorc1 Activation in Natural Killer Cells. J Biol Chem (2019) 294(12):4644–55. doi: 10.1074/jbc.RA118.005892 PMC6433059 30696773

[12] Sabatini DM. Twenty-Five Years of mTOR: Uncovering the Link From Nutrients to Growth. Proc Natl Acad Sci USA (2017) 114(45):11818–25. doi: 10.1073/pnas.1716173114 PMC5692607 29078414

[13] Broer S. Amino Acid Transport Across Mammalian Intestinal and Renal Epithelia. Physiol Rev (2008) 88(1):249–86. doi: 10.1152/physrev.00018.2006 18195088

[14] Galietta LJ Musante L Romio L Caruso U Fantasia A Gazzolo A. An Electrogenic Amino Acid Transporter in the Apical Membrane of Cultured Human Bronchial Epithelial Cells. Am J Physiol (1998) 275(5):L917–23. doi: 10.1152/ajplung.1998.275.5.L917 9815109

[15] Sloan JL Grubb BR Mager S. Expression of the Amino Acid Transporter ATB 0+ in Lung: Possible Role in Luminal Protein Removal. Am J Physiol Lung Cell Mol Physiol (2003) 284(1):L39–49. doi: 10.1152/ajplung.00164.2002 12388375

[16] Di Paola M Park AJ Ahmadi S Roach EJ Wu YS Struder-Kypke M. SLC6A14 Is a Genetic Modifier of Cystic Fibrosis That Regulates Pseudomonas Aeruginosa Attachment to Human Bronchial Epithelial Cells. mBio (2017) 8(6):e02073-17. doi: 10.1128/mBio.02073-17 PMC5736915 29259090

[17] Rotoli BM Visigalli R Barilli A Ferrari F Bianchi MG Di Lascia M. Functional Analysis of OCTN2 and ATB0,+ in Normal Human Airway Epithelial Cells. PloS One (2020) 15(2):e0228568. 32027707 10.1371/journal.pone.0228568 PMC7004352

[18] Gutierrez-Vazquez C Quintana FJ. Regulation of the Immune Response by the Aryl Hydrocarbon Receptor. Immunity (2018) 48(1):19–33. doi: 10.1016/j.immuni.2017.12.012 29343438 PMC5777317

